# Paclitaxel Plus Cetuximab as 1st Line Chemotherapy in Platinum-Based Chemoradiotherapy-Refractory Patients With Squamous Cell Carcinoma of the Head and Neck

**DOI:** 10.3389/fonc.2018.00339

**Published:** 2018-08-27

**Authors:** Tomohiro Enokida, Susumu Okano, Takao Fujisawa, Yuri Ueda, Shinya Uozumi, Makoto Tahara

**Affiliations:** ^1^Department of Head and Neck Medical Oncology, National Cancer Center Hospital East, Kashiwa, Japan; ^2^Division of Pharmacy, National Cancer Center Hospital East, Kashiwa, Japan

**Keywords:** paclitaxel, cetuximab, chemoradiotherapy, platinum-refractory, squamous cell carcinoma of the head and neck

## Abstract

**Purpose:** We sought to evaluate the efficacy and safety of the combination of cetuximab (Cmab) and paclitaxel (PTX) in patients with squamous cell carcinoma of the head and neck (SCCHN) who had unresectable recurrent or metastatic (R/M) disease after platinum-based chemoradiotherapy.

**Materials and Methods:** Data on 23 patients with SCCHN who received paclitaxel and cetuximab (Cmab) for R/M disease no more than 6 months after CRT completion were retrospectively reviewed. PTX and Cmab were given in a 28-day cycle (PTX, 80 mg/m^2^ on days 1, 8, and 15; Cmab, loading dose 400 mg/m^2^ followed by a weekly 250 mg/m^2^). The differences in prognosis between subgroups in different clinical settings were also assessed.

**Results:** CRT had been delivered as definitive treatment in 13 cases (57%) and as adjuvant treatment in 10 (43%). Median time from CRT completion to disease recurrence or metastasis was 73 days (1–152). The best objective response and disease control rates were 52 and 83%, respectively, with 12 partial responses and seven cases of stable disease by Response Evaluation Criteria in Solid Tumors (RECIST). A total of 17 of 23 patients (74%) achieved a degree of tumor shrinkage. Median progression-free survival (PFS) and overall survival (OS) were 7.0 (95% confidence interval [CI]: 3.7–8.4) and 16.3 months (95% CI: 7.8–23.3), respectively. Patients with a longer duration (≥60 d) from CRT completion to disease progression had a statistically significantly longer OS than the others (median OS 22.3 vs. 8.1 months, log-rank test; *p* = 0.034). Main Grade 3 toxicities included neutropenia (13%), anemia (13%), and hypomagnesemia (13%). No Grade 4 toxicity or treatment-related death was seen.

**Conclusion:** PTX and Cmab is a tolerable and effective option in SCCHN patients with symptomatic CRT-refractory disease. Its favorable effects on tumor shrinkage will help relieve tumor-associated symptoms.

## Introduction

Head and neck cancer is the sixth-most common cancer worldwide, and more than 600,000 new cases of squamous cell carcinoma of the head and neck cancer (SCCHN) are diagnosed annually (1, 2). Optimal management of these patients requires a multidisciplinary approach involving radiation oncologists, medical oncologists, and head and neck surgeons. Chemoradiotherapy (CRT) plays an important role in the treatment of head and neck cancer as both a definitive treatment as well as post-operative adjuvant treatment (3–6). However, the recurrence rate of stage III/IV disease after curative or post-operative adjuvant chemoradiotherapy is about 30–40% in the first 2 years of follow up (5–7). For these patients, treatment options are scarce and survival is dismal. In unresectable recurrent or metastatic (R/M) disease after chemoradiotherapy, palliative chemotherapy is the mainstay of treatment. Patients who progress relatively early in their disease course after the last administered dose of a platinum agent (within 6 months as a general guide) have been referred to as “platinum-refractory.” Retreatment with platinum in the setting of platinum-refractory disease has been shown to increase toxicity without improving outcome (8, 9), and it is commonly understood that these patients should be treated with a non-platinum-containing regimen after that date.

As preclinical studies have shown that the combination of cetuximab (Cmab) and taxanes seems to be synergistic (10, 11), paclitaxel (PTX) plus Cmab is a palliative option after failure of platinum-based therapy, offering overall response rates (ORRs) of 38–55% and median OS of 7.6–10 months (12–14). Among others, Hitt et al. prospectively showed that PTX and Cmab was active (ORR54%, median PFS 4.2months, median OS 8.1 months) as 1st line treatment in R/M HNSCC patients, for whom platinum is contraindicated (15). Nevertheless, data on PTX and Cmab as 1st line treatment in patients with platinum-based CRT-refractory SCCHN is lacking. This is the first report to focus on the efficacy and safety of PTX and Cmab in patients with highly aggressive disease, who we often experience in daily practice. In addition, several factors have been considered to be potentially prognostic in head and neck cancer patients who relapse after curative treatment [e.g., clinical setting of CRT [definitive vs. adjutant] (16) or recurrence pattern (17)]. Furthermore, Cmab-containing regimens may provide different clinical activity according to the primary site (18). Accordingly, we attempted to evaluate primary site as predictive factor of PTX and Cmab in subgroup analyses.

## Materials and methods

### Patient population

To extract a heterogeneous population of platinum-based CRT-refractory patients who received PTX and Cmab as 1st line treatment, we reviewed data for 74 consecutive patients with histologically proven head and neck cancer treated with PTX and Cmab between December 2012 and October 2017 at the National Cancer Center Hospital East, Japan. After the selection process, which included excluding patients with prior exposure to either PTX or Cmab as part of induction or definitive treatment, the final analysis was restricted to those 23 patients with SCCHN who received a combination of PTX and Cmab as 1st line treatment for recurrent or metastatic disease no more than 6 months after platinum-based CRT completion (Supplementary Figure 1). They were therefore assumed to be platinum-refractory. Data on patient demographics, tumor characteristics, treatment-related toxicities, and responses were collected. The study was reviewed and approved by the institutional review board.

### Treatment

All patients were required to have adequate hematological, hepatic and renal function before treatment. PTX and Cmab were given in a 28-day cycle, with PTX administered weekly at a dose of 80 mg/m^2^ over 1 h on days 1, 8, and 15 of each cycle. Cmab was administered at a loading dose of 400 mg/m^2^ during a 2-h infusion, followed by a weekly 1-h infusion of 250 mg/m^2^ on days 1, 8, 15, and 22 of the treatment cycle. Some patients were switched at the completion of six cycles of PTX and Cmab to Cmab maintenance therapy at the discretion of the attending physician. Patients received Cmab monotherapy as a maintenance therapy until disease progression or until unacceptable toxic effects. All patients were premedicated with 13.3 mg of dexamethasone, 50 mg of ranitidine, and 8 mg of ondansetron before each dose of PTX and Cmab. Dexamethasone 6.6 mg and chlorpheniramine (H1 blocker) 5 mg were given on the days of Cmab monotherapy.

### Evaluation of efficacy and toxicity

Clinical response to treatment was evaluated radiographically using computerized tomography imaging approximately every 8 weeks. Anti-tumor activity was retrospectively evaluated according to the Response Evaluation Criteria in Solid Tumors (RECIST) v.1.1 via the review of imaging results. Toxicity during treatment was graded using the Common Toxicity Criteria for Adverse Event (CTCAE version 4.0).

### Statistical analysis

Progression-free survival (PFS) and Overall survival (OS) were calculated by the Kaplan-Meier product-limit method. The end of PFS was defined as disease progression or death from any cause, while the end of OS was determined as death from any cause. All other events were censored. Hazard ratios (HRs) were calculated by Cox regression analysis. The differences in PFS and OS between patients with oral cavity cancer and others, the differences between patients who received CRT as definitive treatment and as adjuvant treatment, and the differences between patients with and without metastatic disease were assessed using stratified log-rank tests. Statistical analyses were two-tailed and were performed using Prism version 6 software (GraphPad Software, Inc., La Jolla CA, USA). A *p*-value >0.05 was considered statistically significant.

## Results

### Patient characteristics

Characteristics of the 23 eligible patients are summarized in Table 1. Most patients were men (87%), and median age was 65 year (range 35–74 year). All patients had undergone radiotherapy and concurrent cisplatin (CDDP), delivered as definitive treatment in 13 cases (57%) and as adjuvant treatment in 10 (43%).

**Table 1:** Patient characteristics.

**Characteristic**	**Patients**, ***n*** **(%)**	
**Age [year]**		
Median (range)	65 (35–74)	
**Gender**		
Male	20	(8)
Female	3	(13)
**ECOG performance status**		
0	6	(26)
1	17	(74)
**Primary site**		
Oral cavity	10	(43)
Hypopharynx	7	(30)
Oropharynx	3	(13)
Larynx	1	(4)
Unknown primary	2	(9)
**Smoking [pack-years]**		
Median (range)	30	(0–128)
**Clinical setting of chemoradiotherapy**		
Definitive chemoradiotherapy	13	(57)
Adjuvant chemoradiotherapry	10	(43)
**Cumulative CDDP dose during CRT [mg/m**^2^**] Median (range)**		
IV	240	(80–300)
IA	700	(700)
**Radiotherapy dose during CRT [Gy]**		
Median (range)	66	(50–70)
**Time from chemoradiothrerapy to recurrence or metastasis [days]**		
Median (range)	73	(1–152)
**Disease status at PTX** + **Cmab initiation**		
Loco-regional only	7	(30)
Distant only	7	(30)
Both loco-regional and distant	9	(40)

*CRT, chemoradiotherapy; CDDP, cisplatin; PTX, paclitaxel; Cmab, cetuximab; IV, intravenous infusion; IA, intra-arterial infusion*.

### Treatment and efficacy

**Figure 1:**
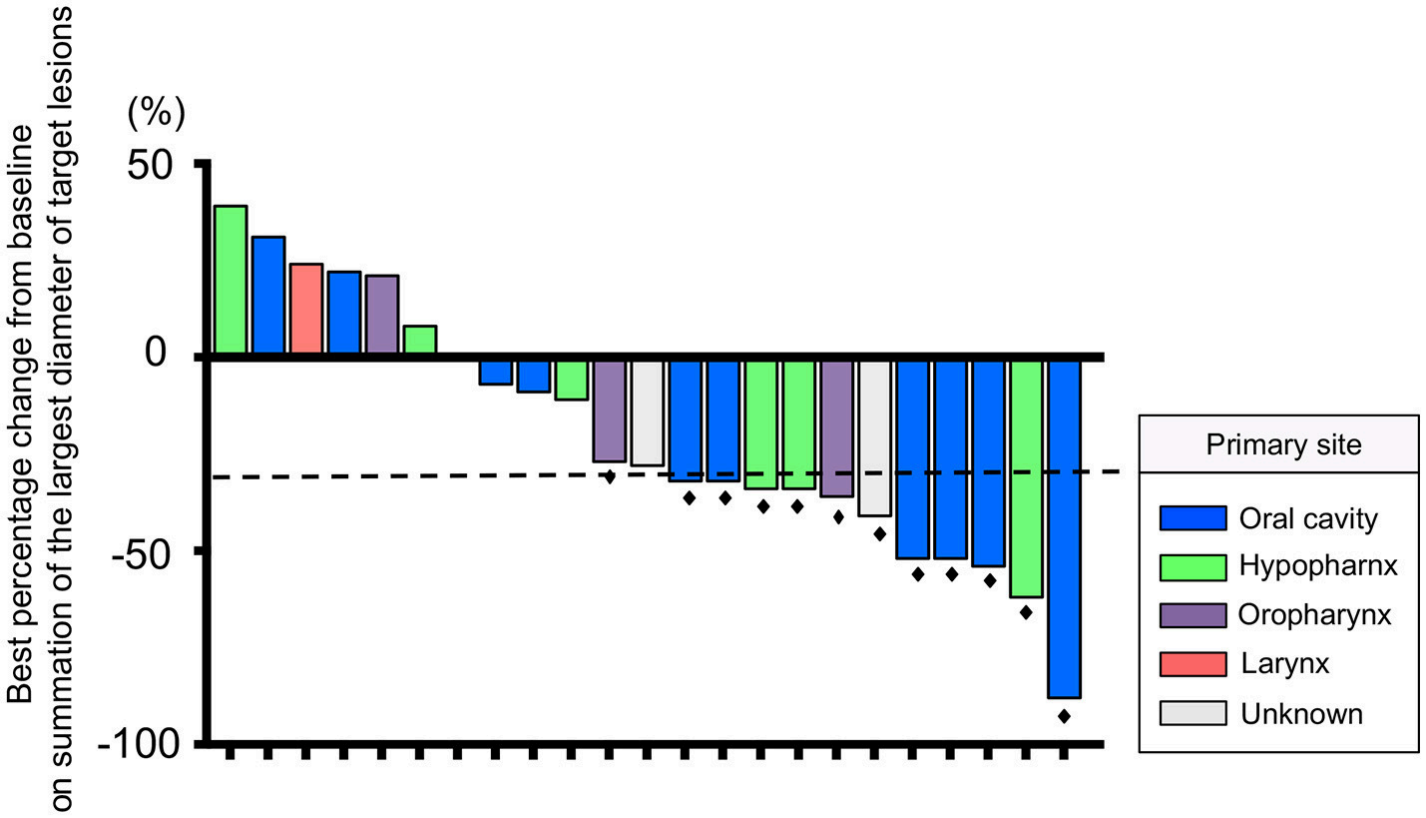
Waterfall plot of the maximum percentage change from baseline on summation of the largest diameter of target lesions for 23 patients. The *dashed line* indicates a 30% reduction in tumor burden in the target lesion. *Black dots* indicate patients who had a response according to RECIST version 1.1.

The median number of administrations given was 12 (range: 4–35) for PTX and 18.5 (range: 5–46) for Cmab. Eight patients (35%) proceeded to Cmab maintenance therapy. Among them, physicians decided to switch four patients to Cmab maintenance at the completion of six cycles of PTX and Cmab. Three patients experienced unacceptable PTX-induced toxicity, and discontinued PTX at that time, moving to Cmab maintenance. The majority of patients, 77%, began other chemotherapy after discontinuation of PTX and Cmab (Table 2). With a median follow up of 12.9 months (range 3.6–42.9), objective overall response (ORR) and disease control rate (DCR) was 52% (95% confidence interval [CI] 33–71%) and 83% (95% CI 62–94%), respectively. Twelve patients had partial responses (PR)(52%) and seven had stable disease (30%) (Table 3). Best percent change in tumor diameter (maximum lengths of all target lesions in the patient) were summed and change in tumor burden over time are shown in Figure 1.

**Table 2:** Summary of treatment.

**Characteristic**	**Patients**, ***n***	
**Number of PTX administrations**		
Median (range)	12	(4–35)
**Number of Cmab administrations**		
Median (range)	18.5	(5–46)
**Cmab maintenance therapy (%)**		
No	15	(65)
Yes	8	(35)
**Reason for proceeding to maintenance therapy**		
Physicians' decision at the completion of 6 cycles of paclitaxel and cetuximab	4	(17)
PTX induced unacceptable toxicity*	3	(13)
Patient preference	1	(4)
**Number of Cmab administrations as maintenance therapy**		
Median (range)	6	(3–61)
**Reason for discontinuation of PTX**+ **Cmab**^†^ **(%)**		
Progressive disease	20	(91)
Performance status worsened	1	(5)
Surgery	1	(5)
**Subsequent treatment of PTX** + **Cmab**^†^ **(%)**		
None	3	(14)
Chemotherapy	17	(77)
Radiotherapy	1	(5)
Surgery	1	(5)

PTX, paclitaxel; Cmab, cetuximab;

* *Grade 2 malaise in all three patients. Out of 22 patients who failed treatment of PTX + Cmab at cutoff date*.

**Table 3:** Best response by treatment^†^.

**Characteristic**	**Patients**, ***n*** **(%)**	
CR	0	(0)
PR	12	(52)
SD	7	(30)
PD	4	(17)
Overall response rate (95%CI)	52% (33–71)	
Disease control rate (95%CI)	83% (62–94)	

CR, complete response; PR, partial response; SD, stable disease; PD, progressive disease.

† *RECIST v. 1.1*.

**Figure 2:**
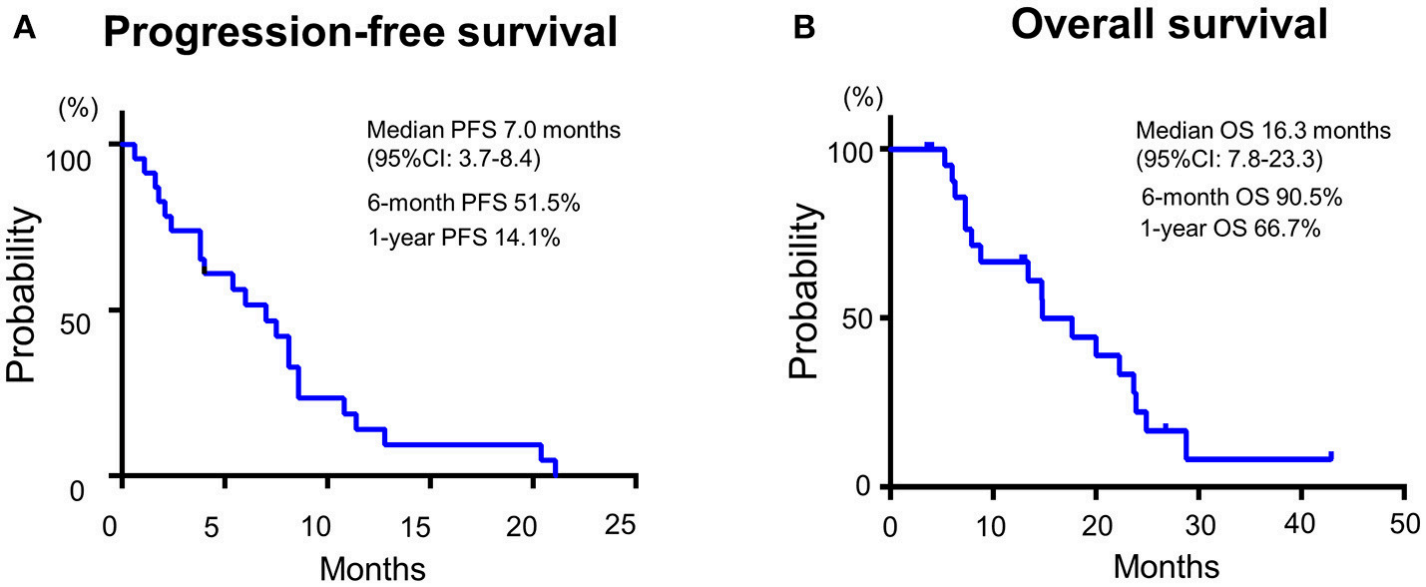
Patient **(A)** progression-free survival and **(B)** overall survival of SCCHN patients with platinum-based CRT-refractory R/M disease treated with the combination of PTX and Cmab in 1st line setting.

**Figure 3:**
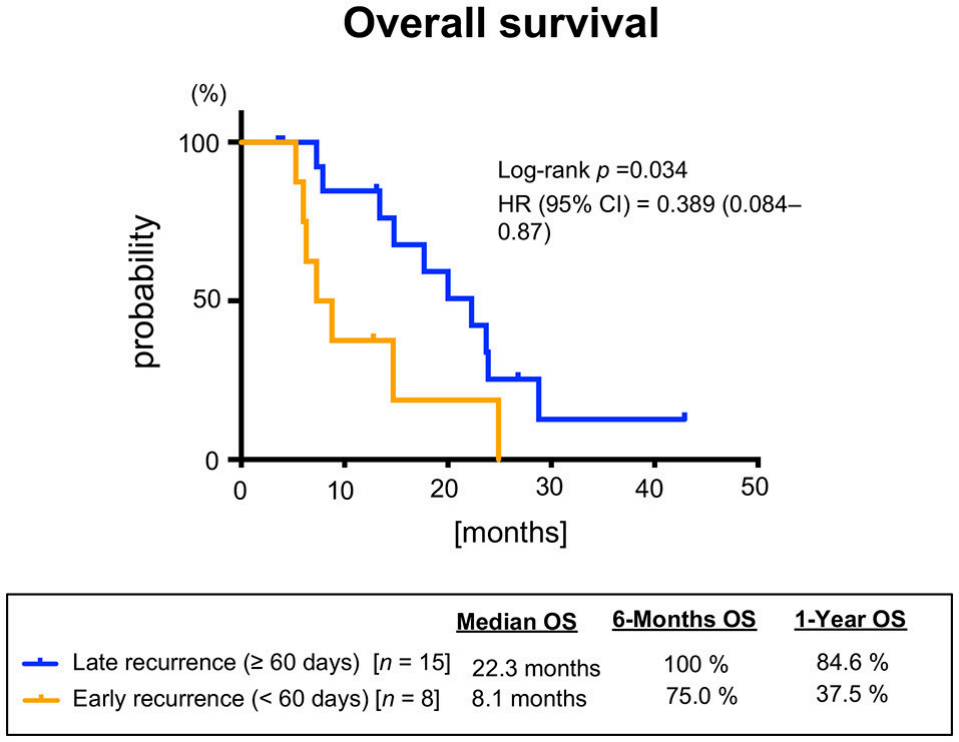
Overall survival stratified according to the interval between chemoradiotherapy and recurrence or metastasis.

Median PFS and OS were 7.0 (95%CI: 3.7–8.4) and 16.3 months (95%CI: 7.8–23.3), respectively (Figure 2). Additionally, we observed a trend toward improved PFS and a statistically significantly favorable OS in patients with longer duration (≥60 days) from CRT completion to disease recurrence or metastasis (Figure 3 and Supplementary Figure 2). There were no apparent differences in response or prognosis according to clinical setting of CRT (definitive vs. adjuvant), primary site (oral cavity vs. others) or presence or absence of locoregional disease (Supplementary Figure 3).

**Figure 4:**
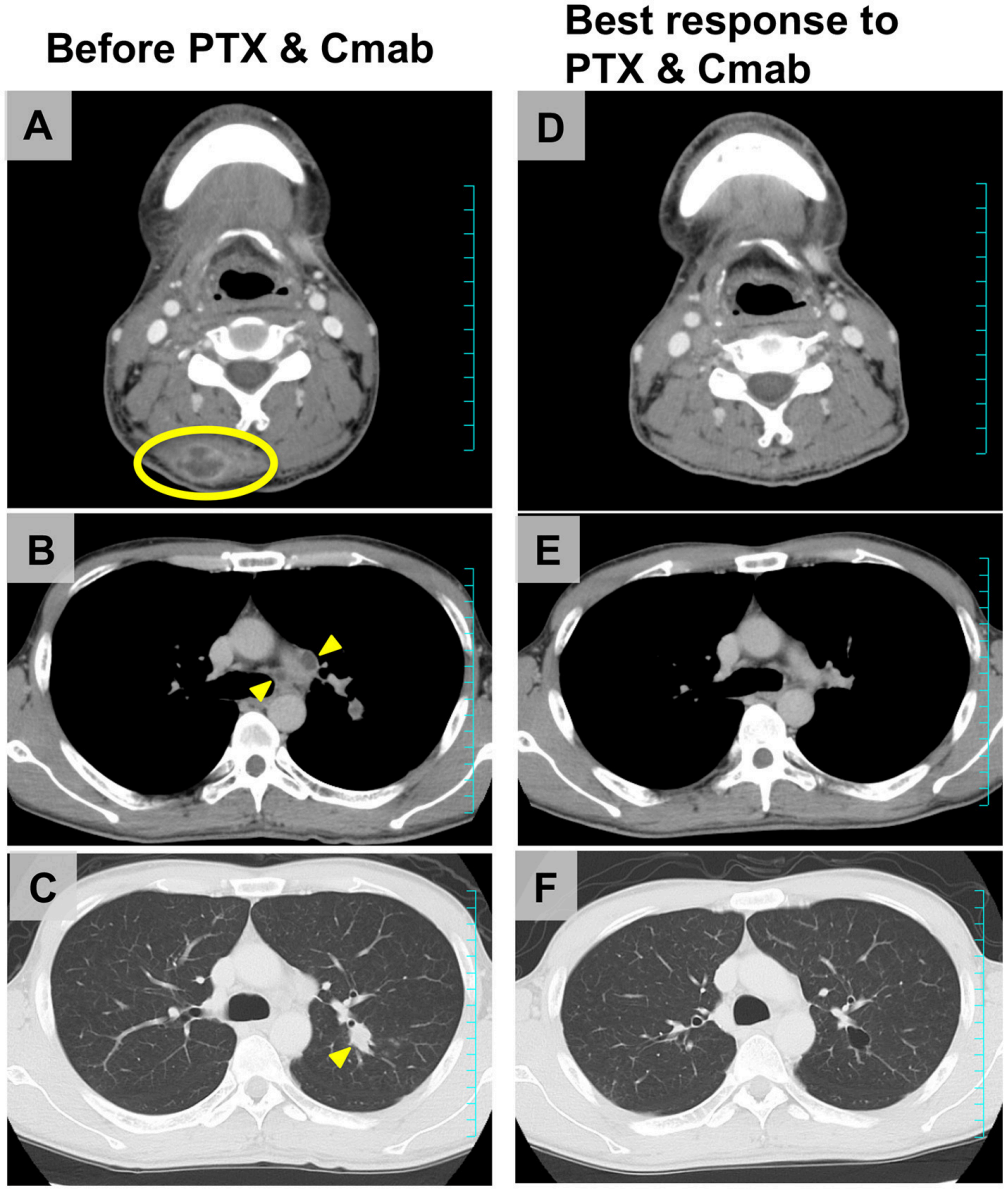
Representative imaging from a tongue squamous cell carcinoma patient who achieved a favorable clinical response after CRT failure, a male initially treated with partial glossectomy and neck dissection and adjuvant CRT. **(A–C)** The tumor recurred in the trapezius (yellow ellipse), mediastinal lymph nodes, and lung (yellow arrowheads) 5 months after completion of CRT. **(D–F)** After four cycles of therapy (PTX 80 mg/m^2^, days 1, 8, and 15; and Cmab, 400 mg/m^2^ followed by a weekly 250 mg/m^2^; 28-day cycle), almost all recurrent lesions had disappeared and occipital pain was completely alleviated.

Figure 4 shows scans of a tongue cancer patient with recurrent disease located in the trapezius, mediastinal lymph nodes, and lung, 5 months after completion of post-operative adjuvant chemoradiotherapy (cumulative CDDP dose: 200 mg/m^2^ plus radiotherapy: 66 Gy) (Figures 4A–C). After one cycle of PTX and Cmab, his tumor-associated occipital pain was significantly relieved. Following three cycles, almost all recurrent lesions had disappeared (Figures 4D–F). We then switched from PTX and Cmab to Cmab monotherapy according to the patient's preference; vertebral metastases appeared 1 month after. He eventually received PTX and Cmab (6 months) and subsequent Cmab monotherapy (1 month) for a total of 7 months.

### Toxicity

Adverse events observed are listed in Table 4. Two patients (13%) developed Grade 3 anemia and required blood transfusions. Three patients (13%) developed Grade 3 neutropenia. No patient developed thrombocytopenia or febrile neutropenia of any grade. The most common non-hematological toxicity was skin toxicities (acneiform dermatitis, paronychia, skin cracks, and dry skin), which variously occurred in 20 patients (95%). The second-most common non-hematological toxicity was neuropathy, which was documented in 17 (74%) patients. Prolonged Grade 2 malaise was the stated reason for PTX discontinuation in three patients, who then proceeded to Cmab maintenance therapy. Although one patient developed Grade 3 septicemia and another experienced Grade 3 pulmonary embolism during treatment, they fully recovered. Hypomagnesemia was observed in 14 (67%) patients, and was Grade 3 in 3 patients (13%). No patient experienced Grade 4 toxicity, and no treatment-related deaths were seen.

**Table 4:** Summary of adverse events.

**Toxicity**	**All grades Patients, *n* (%)**	**Grade 3 Patients, *n* (%)**
**HEMATOLOGIC**		
Leukocytopenia (%)	21 (91)	5 (24)
Neutropenia (%)	17 (74)	3 (13)
Anemia (%)	19 (83)	2 (9)
Thrombocytopenia (%)	0 (0)	0 (0)
Febrile neutropenia (%)	–	–
**NONHEMATOLOGIC**		
AST increased (%)	3 (13)	0 (0)
ALT increased (%)	7 (30)	0 (0)
Acute kidney injury (%)	1 (5)	0 (0)
Hypomagnesemia (%)	14 (67)	3 (13)
Hyperglycemia (%)	1 (5)	1 (5)
Proteinuria (%)	1 (5)	1 (5)
Peripheral sensory neuropathy (%)	17 (74)	0 (0)
Malaise (%)	12 (57)	0 (0)
Arthralgia (%)	3 (13)	0 (0)
Constipation (%)	3 (13)	0 (0)
Mucositis (%)	7 (33)	0 (0)
Dysgeusia (%)	5 (24)	–
Acneiform dermatitis (%)	14 (67)	3 (13)
Paronychia (%)	12 (57)	1(5)
Skin cracks (%)	15 (71)	0 (0)
Dry skin (%)	16 (76)	0 (0)
Blood stream infection (%)	1 (5)	1 (5)
Thromboembolic event^†^ (%)	1 (5)	1 (5)

*Pulmonary embolism*.

## Discussion

The outcome of patients with recurrent and/or metastatic head and neck cancer refractory to platinum-based chemotherapy is unfavorable when treated with conventional chemotherapy alone, with median OS of only around 100 days (19). The results of this study are relatively favorable when compared with other recent studies, which reported median OS of 9.1–10 months (12–14). Reasons for the longer response duration in this study may be that the other studies included patients who received PTX and Cmab as ≥2nd line chemotherapy for recurrence or metastatic disease, and who had had a previous treatment history with PTX, docetaxel (DTX), or Cmab. Moreover, we focused here on platinum-tolerant but platinum-based CRT-refractory patients, who were not a focus of Hitt's study (15). Accordingly, our present study may more accurately reflect the efficacy of PTX and Cmab as 1st line chemotherapy against platinum-based CRT-refractory disease. Values for cumulative CDDP dose during CRT in the present study was sufficient to determine that the cases were truly platinum-refractory.

Until now, there have been few data about the prognosis of patients failing CRT with curative intent. The median overall post-failure survival of patients with loco-regional failure after intensity modulated radiotherapy with/without chemotherapy was 9.37 months (20). Of these patients, a significantly worse prognosis was noted in those unable to undergo salvage surgery (7.4 months vs. 22.6 months; *p* = 0.003). Even though the majority of subjects (95%) in our study had not undergone salvage surgery after CRT failure, median OS was more than double (16.3 months), which suggests the promising efficacy of PTX and Cmab for platinum-refractory SCCHN.

The agent that competes with the treatment regime in our study is the anti-PD-1 antibody, nivolumab. CheckMate 141 was a phase III trial that enrolled 361 patients with R/M SCCHN, of any tumor PD-L1 expression status, who had disease progression within 6 months after platinum-based chemotherapy (21). This trial compared nivolumab to the investigators' selected standard therapy, namely methotrexate, DTX, or Cmab. Nivolumab monotherapy provided a longer OS than standard therapy, with a median OS of 7.5 vs. 5.1 months for standard therapy. Further, ORR was 13.3% for nivolumab vs. 5.8% for standard therapy. Outcomes from Checkmate 141 among patients whose disease was platinum-refractory in the primary or adjuvant setting and who received nivolumab or the investigators' selected treatment as 1st line therapy for R/M have been presented (22). In this situation, ORR, median PFS and OS in the nivolumab arm were 19.2%, 2.3 months, and 7.7 months, respectively. Among Asian patients in the CheckMate 141 study, nine of 23 patients (39%) in the nivolumab group experienced a degree of tumor shrinkage and ORR was 26.1% by RECIST. In contrast, 17 of 23 patients (74%) receiving PTX and Cmab in our study experienced tumor shrinkage and ORR was 52% by RECIST. Our findings suggest that PTX and Cmab may offer comparable or greater anti-tumor activity than nivolumab, especially in terms of tumor shrinkage, which may benefit patients with significant tumor-associated symptoms, as seen in Table 3 and Figure 4. However, we should also note that these are unadjusted non-comparative descriptive data from a small numbers of patients. Further prospective evaluation of this combination within this population is warranted.

An important aspect of palliative chemotherapy includes improvement or maintenance of quality of life (QoL). Although we did not assess the QoL in these patients, three patients (13%) switched to Cmab maintenance therapy from PTX and Cmab combination because of general malaise thought to be due to PTX. Immune checkpoint inhibitors, including nivolumab, generally provide favorable QoL profiles when compared with conventional chemotherapy or molecular targeted drugs (21). It is important that agent selection be appropriate to the situation of the individual patient, such as the necessity or otherwise of prompt tumor shrinkage, in order to achieve maximum benefit with favorable QoL.

Several limitations of this study should be mentioned. First, our study was retrospective and without a control arm. It would therefore be interesting to perform a similar analysis in a cohort of patients treated with other drugs (e.g., Nivolumab) in the same setting as described above. Second, while the eligibility review process indeed provided heterogeneous population, this eventually resulted in a small number of enrolled patients for final analysis. Accordingly, our results should be evaluated with particular care, especially those of the subgroup analysis, which warrant further investigation.

## Conclusion

In this work, we demonstrated that PTX and Cmab is a tolerable and effective option in SCCHN patients with platinum-based CRT-refractory disease. Its favorable effects on tumor shrinkage may help relieve tumor-associated symptoms.

## Author contributions

TE participated in the study concept and design, interpreted the data, and drafted the manuscript. MT extracted, managed, and analyzed the data. All authors provided critical revisions and approved the final manuscript.

### Conflict of interest statement

SO and MT receive honoraria from Merck Serono.

The remaining authors declare that the research was conducted in the absence of any commercial or financial relationships that could be construed as a potential conflict of interest.
