# Supplementary figures and images for: Paclitaxel Plus Cetuximab as 1st Line Chemotherapy in Platinum-Based Chemoradiotherapy-Refractory Patients With Squamous Cell Carcinoma of the Head and Neck
Source: Front Oncol. 2018 Aug 27;8:339. doi: 10.3389/fonc.2018.00339 (PMC6119881; doi:10.3389/fonc.2018.00339)

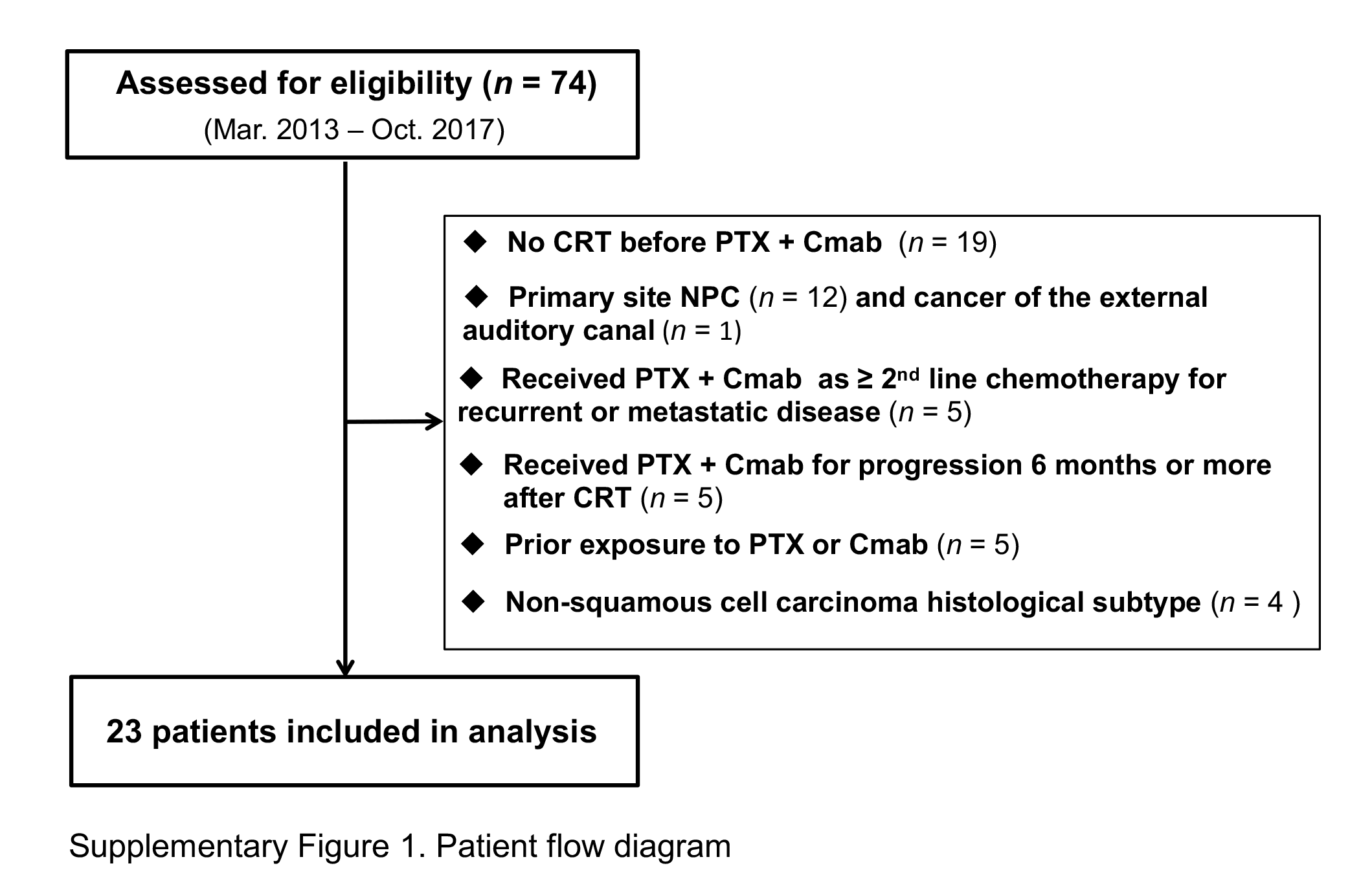

Supplement: Supplementary file 1 [file Image_1.TIFF]

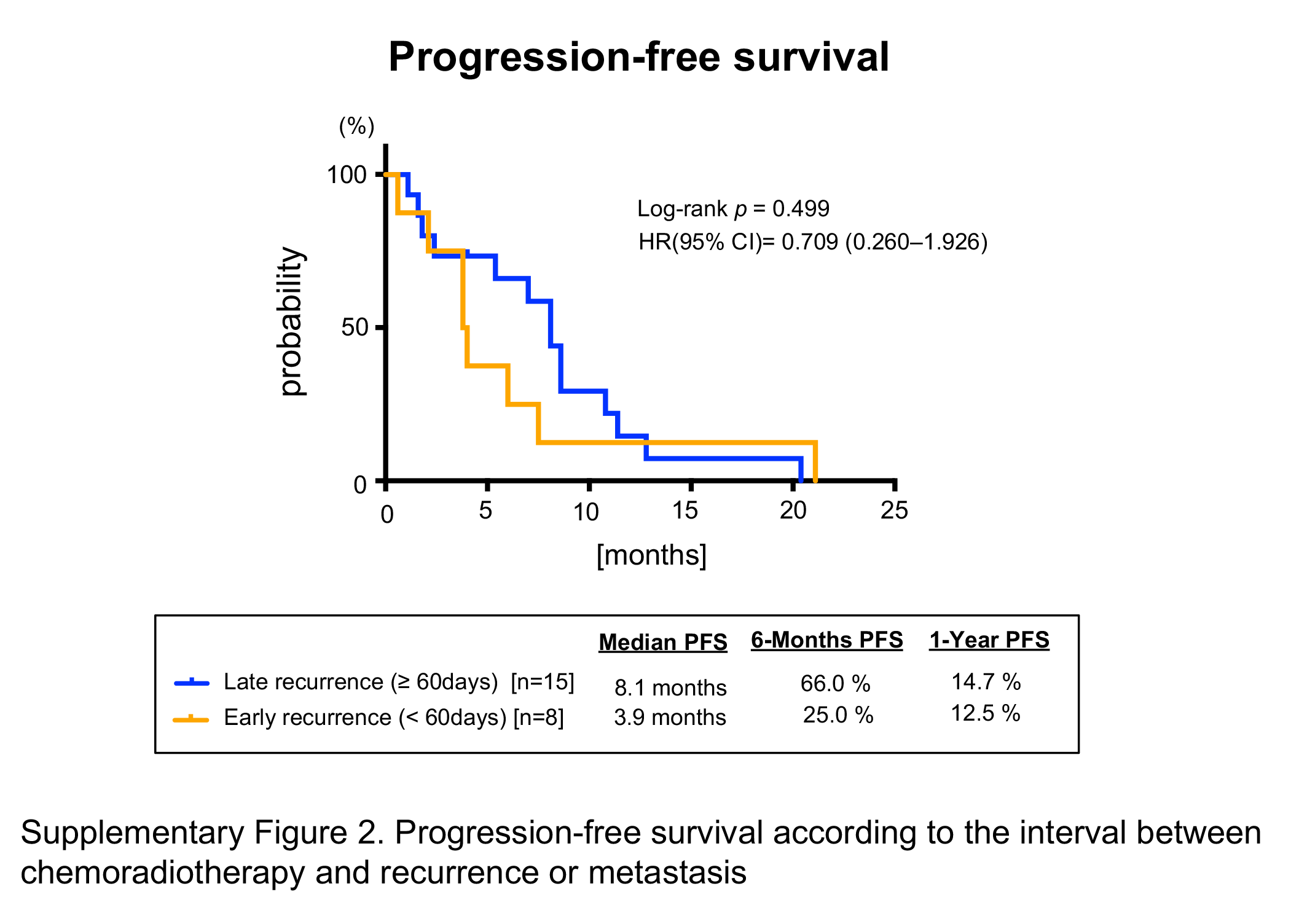

Supplement: Supplementary file 2 [file Image_2.TIFF]

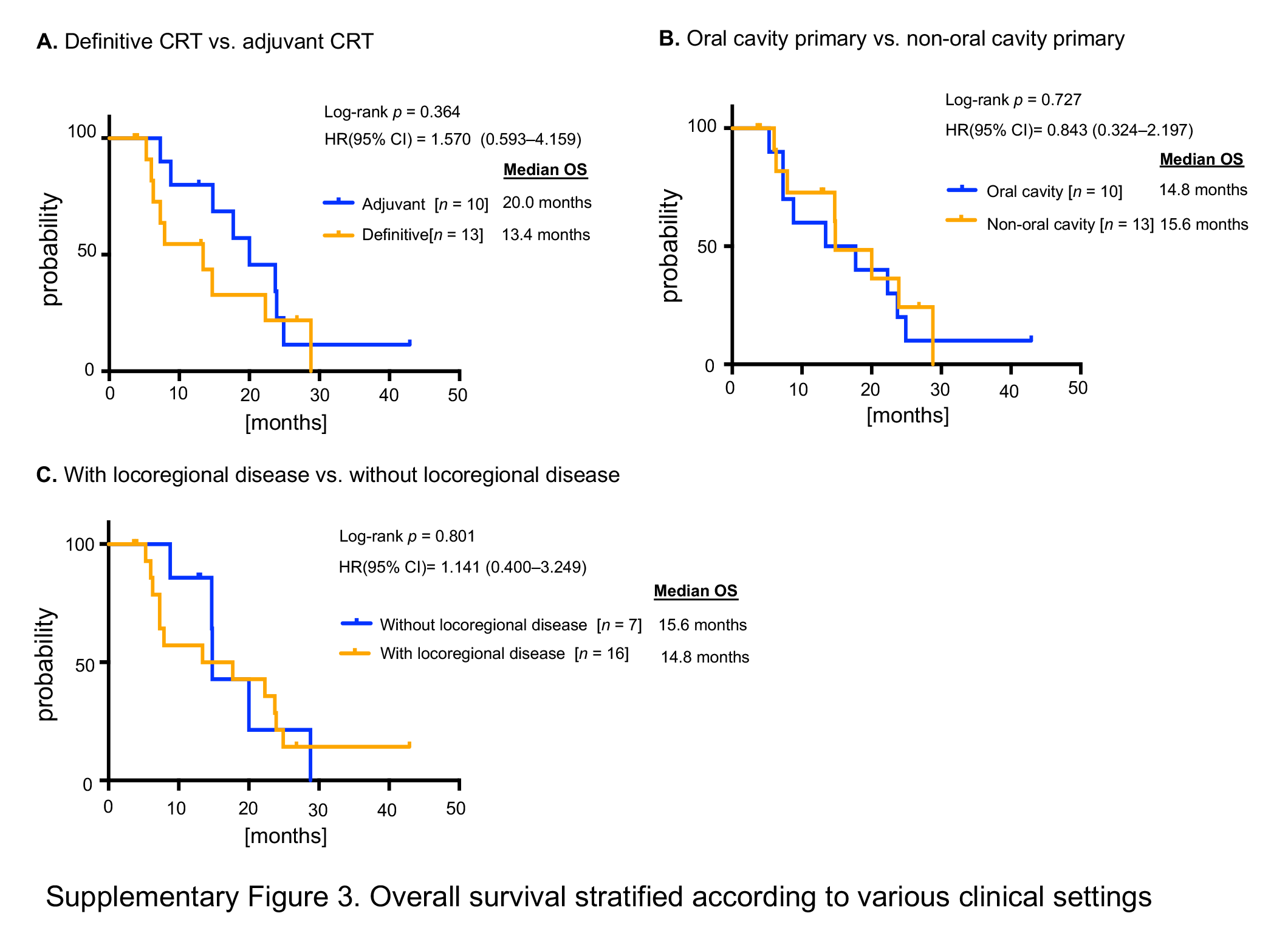

Supplement: Supplementary file 3 [file Image_3.TIFF]
